# Assessing health-related quality of life of Chinese population using CQ-11D

**DOI:** 10.1186/s12955-024-02250-1

**Published:** 2024-04-19

**Authors:** Jie Pan, Qianxi Han, Pingda Zhou, Jiameng Zhou, Mengpei Zhang, Wentao Zhu

**Affiliations:** 1 School of Traditional Chinese Medicine, Beijing University of Chinese Medicine, Beijing, China; 2 School of Management, Beijing University of Chinese Medicine, Beijing, China; 3 University of Chinese Medicine, Higher education zone in LiangXiang Town, FangShan District, Beijing, 102401 China

**Keywords:** Health-related quality of life, CQ-11D, China

## Abstract

**Purpose:**

This study aimed to assess the health-related quality of life (HRQoL) of the Chinese population by using the Chinese medicine quality of life-11 dimensions (CQ-11D) questionnaire and to identify factors associated with HRQoL.

**Methods:**

The data was derived from a survey conducted by the Institute of Pharmacoeconomics Evaluation at Beijing University of Chinese Medicine on the quality of life of the Chinese population. The sex and age of respondents were considered through quota sampling. Demographic, socioeconomic, and health indicators were collected using the structured questionnaire. We performed bivariate analyses first to examine the associations between the above factors and the HRQoL of respondents measured by the CQ-11D. Multivariate linear regression and ordinal logistic regression models were established to analyze the factors (demographic, socioeconomic, and health indicators) differences in HRQoL, as well as the risk of each group reporting problems across the 11 dimensions of CQ-11D.

**Results:**

From February 2021 to November 2022, a total of 7,604 respondents were involved and 7,498 respondents were included. The sample approximated the general adult Chinese population in terms of age, sex, and district of residence, and each geographic distribution ranged from 9.71 to 25.54%. Of the respondents, 45.84% were male, and 89.82% were Han ethnicity. The mean utility score ranged from 0.796 to 0.921 as age increased. According to the respondents, most health problems were identified in the PL (fatigue) (70.16%) and SM (quality of sleep) (63.63%) dimensions. The CQ-11D index scores varied with the demographic and socioeconomic characteristics of respondents, except for ethnicity (*p* > 0.05) and income (*p* > 0.05). The multivariate analysis revealed significant negative associations between health utility scores and various factors. These factors include sex (female), age over 65, belonging to ethnic minorities, rural household registration, being widowed or divorced, having a primary school education or below, being a student or unemployed, having a low income of 0–1,300, engaging in smoking or drinking, limited participation in physical activities, experiencing changes in self-perceived health status compared to the previous year, and having chronic diseases. The odds of respondents reporting problems in 11 dimensions varied among different socio-demographic groups.

**Conclusions:**

This study reports the first Chinese population norms for the CQ-11D derived using a representative sample of the Chinese general population. Self-reported health status measured by the CQ-11D varies among different socio-economic groups. In addition to participation a physical activity and the presence of chronic disease, smoking and drinking also significantly influence HRQoL.

**Supplementary Information:**

The online version contains supplementary material available at 10.1186/s12955-024-02250-1

## Introduction

Quality of life ( QoL ) is defined as “an individual’s perception of their position in life in the context of the culture and value systems in which they live and in relation to their goals, expectations, standards, and concerns” [1]. QoL includes solving complex, multi-factor relationships, covering a wide range of economic, socio-cultural, and lifestyle factors. With the integration of quality of life (QoL) and medical practice, the concept of Health-Related Quality of Life (HRQoL) has emerged [2]. The term “Health-Related Quality of Life” (HRQoL) is used to define an individual’s specific perception of health or to solely represent the utility associated with health conditions [3, 4], which is a subset of overall quality of life (QoL). QoL measures are valuable for clinical studies for several reasons, which was used to quantify the impact of a condition and to compare the effects of disease or used to evaluate changes resulting from therapeutic intervention or the course of disease [5]. Besides, QoL measures are necessary as a central component of cost-utility analysis (CUA), which has been widely used in health technology assessment and health policy decisions [6, 7].

Over the past few decades, several HRQoL assessment instruments have been developed. Generic preference-based measures (GPBMs) such as the EuroQol five-dimension (EQ-5D) [8] and the Short Form six-dimension (SF-6D) survey [9–11] have become widely accepted in health utility studies. The EQ-5D has been developed in 2 versions with the same descriptive system and comprises five dimensions: mobility, self-care, usual activities, pain/discomfort, and anxiety/depression [12, 13]. The health state classification system of SF-6Dv2 comprises six dimensions: physical functioning, role limitation, social functioning, pain, mental health, and vitality [14, 15]. Most of the GPBMs were developed in Europe and North America, and are often translated into other languages to use in many non-English speaking countries [16, 17]. However, health is a culturally related concept, and health evaluation indicators formulated in the Western cultural environment may not include Chinese cultural views on health [18]. GPBMs focus on general health status, including physical, functional, and emotional domains. Currently, previous studies have carried out the construction of Chinese population norms based on the EQ-5D and SF-6D [19–25]. The assessment instruments, EQ-5D-5 L and SF-6D, which have been widely used and developed, particularly based on foreign populations, have been found to exhibit ceiling and floor effects when measuring HRQoL [29–31].

The CQ-11D is a measurement tool developed specifically for the Chinese population to assess the quality of life [26, 27]. It is primarily used for evaluating the health utility of Traditional Chinese Medicine interventions as well as the general population’s quality of life. The development of CQ-11D is based on the World Health Organization’s (WHO) concept of quality of life and is guided by principles rooted in traditional Chinese medicine theory and the Chinese perspective on health. The theoretical and methodological framework of CQ-11D draws upon the domestic and international quality of life instruments and health utility scoring systems. Through a combination of literature research, Delphi method expert consultation, and Discrete Choice Experiment with Time Trade-Off (DCE_TTO_) surveys, the items of the instrument have been identified and a corresponding health utility scoring system has been established. Existing research has confirmed the feasibility and good reliability and validity of the CQ-11D instrument [28].

In comparison, the CQ-11D instrument encompasses a greater number of dimensions and more comprehensive categories, offering a broader range of health utility measurements and more comprehensive results. Research has indicated a high level of consistency in the measurement results among these three instruments. Furthermore, the CQ-11D instrument demonstrates higher sensitivity in assessing certain chronic conditions such as hypertension and chronic gastritis [32]. Based on these considerations, this research utilizes the CQ-11D instrument to conduct a comprehensive investigation of the target population to accurately reflect the health preferences and characteristics of the Chinese population.

It is important to note that population health surveys provide comprehensive information about the overall health status of residents as well as longitudinal trends, in addition to supporting the decision-making process in the healthcare field with empirical evidence [21]. Many nations and regions have engaged in extensive research and published population norm data to enhance the utilization of health utility value data pertaining to specific populations in relevant studies. For example, notable contributions in this field have been made by countries such as Japan [33], Brazil [34], and Portugal [35]. Population norms data can be used to compare profiles for patients with particular conditions with data for the average person in the general population from a similar age and sex group [36]. Apart from its utility in capturing disease-specific health states, it can also serve as a comparative tool for assessing the health profiles of patients within subgroups sharing similar age and sex characteristics [37]. Other countries have calculated normative utility scores using the EQ-5D and showed differences between sex, age, education, and other factors [38–41].

Our study aimed to provide population norms for HRQoL in China, based on the CQ-11D questionnaire. In addition, the multiple linear regression model and ordinal logistic regression model were used to explore the association of factors (demographic, socioeconomic, and health indicators) on HRQoL and the differences in 11 dimensions, respectively.

## Methods

### Study design and data collection

Data used in the study was obtained from the quality of life of the Chinese population survey based on the CQ-11D questionnaire, which was conducted by the Institute of Pharmacoeconomics Evaluation of Beijing University of Chinese Medicine [28, 32]. The survey period was from February 2021 to November 2022. To investigate the representativeness of the sample, quota sampling was used in the survey. Quotas were used to account for sex and age group, covering seven districts across the country, and strict training and questionnaire quality control was carried out (details of the survey design have been published [28, 32]). The survey area covered all major cities in seven sub-regions of China, covering seven geographical divisions North China, Northeast China, East China, Central China, South China, Southwest China, and Northwest China. Recruited respondents by posting recruitment advertisements in a way that was convenient for the interviewer. Recruitment was conducted in publicly accessible places (Parks, shops, streets, and university campuses) and private areas (respondents’ residences). A general representative population in China was investigated using one-on-one and face-to-face questionnaire interviews. The main steps of the first survey which was conducted from February 2021 to November 2022 were as follows [28]: The respondents were screened into the research and informed consent; the respondent completed the CQ-11D questionnaire; the respondents completed the DCE_TTO_ tasks. In addition, after completing the DCE_TTO_ tasks, respondents were asked to self-assess the difficulty of understanding and answering these tasks according to a 5-point Likert scale ranging from very easy to very difficult; The respondents answered the background information questionnaire (including the demographic characteristics, socioeconomic status, and health indicator), the EQ-5D-3 L and the SF-6D questionnaires; Recorded the time for the respondent to complete the survey; Checked whether the questionnaire was clear and complete. The second survey was carried out from February to November 2022, including three different survey parts [32]: The respondents were screened into the research and informed consent; The respondents answered the demographic characteristics, socioeconomic status, and health indicator questions; The respondents completed the CQ-11D, EQ-5D-5 L, and SF-6D, respectively. All investigations were conducted with the informed consent of the subjects and with Ethics Committee approval (the ethics committee of the Beijing University of Chinese Medicine, Approval number: 2021BZYLL03012).

For this study, data collected in the background information (the demographic characteristics, socioeconomic status, and health indicators) and CQ-11D questionnaire parts of the survey were utilized.

### Health-related quality of life measured with the CQ-11D

CQ-11D contains 11 items: XD (movement and self-care), SY (appetite), DB (stool), SM (quality of sleep), JS (spirit, including being alive, energetic, and focused), TY (dizziness, including feeling dizzy in the mind, with eyes closed for minor cases, or spinning in front of the scene in serious cases, inability to stand), XH (palpitations, or feeling restless), TT (pain), PL (fatigue), FZ (irritability), JL (anxiety, worried, anxious, nervous, restless), and depression (frustrated, lack of interest in doing things, no fun, low energy) [27]. According to the unity between the body and the Shen (Spirit) theoretical of Chinese medicine, the first 8 dimensions are defined as body dimension (XING) while the last 3 dimensions are defined as Shen dimension (SHEN), as shown in Appendix 1. The health state for each item is categorized into 4 levels of severity (no, slight/occasionally, often, severe), allowing for the description of 4^11^ (i.e., 4,194,304) different health states. The health utility value is calculated based on the item coefficients in the health utility scoring system, with a measurable range of -0.868 to 1. The CQ-11D utility value set can be found in Appendix 1.

### Demographic and health-related variables

Previous studies have shown that there are differences in HRQol among demographic characteristics and socioeconomic status variables [23–25]. Demographic characteristics and socioeconomic status including age, sex, level of education, marital status, ethnicity, occupation, household registration, income per month, and geographical division were collected according to the structured questionnaire. Moreover, lifestyle habits are associated with chronic diseases and may affect quality of life. The interview also collected health indicators on the frequency of participation in sports exercise or fitness activities, drinking, smoking, presence of chronic diseases, and changes in self-perceived health status compared to the previous year.

### Statistical analysis

Demographic and health-related variables were analyzed by estimating mean values and standard deviations (SD) for continuous variables, frequencies, and proportions for categorical variables. We first conducted bivariate analyses to investigate the relationships between the aforementioned factors and the HRQoL of the respondents, as measured by the CQ-11D. Those utilities were compared among the respondents with different characteristics using non-parametric tests (Wilcoxon tests for two categories or Kruskal-Wallis tests for more than two categories) to examine differences in CQ-11D index scores of the respondents because the distribution of data was skewed. The percentage of people reporting any problem in each dimension was calculated and X^2^ tests were performed to determine the statistical significance of the difference between groups in the percentage of reported any problems. The results were presented by sex and age groups.

We used multiple linear regression to examine the associations of socio-demographic characteristics with the CQ-11D index scores. Based on prior knowledge [42–44], covariates included age, sex, education level, marital status, ethnicity, occupation, household registration, income, geographical division, frequency of exercise, smoking behaviors, and presence of chronic conditions. We added drinking behaviors and changes in self-perceived health status compared to the previous year, which may also affect the HRQoL. In addition, ordinal logistic regression was developed with the 11 health dimensions as dependent variables (1 = no, 2 = slight/occasionally, 3 = often, 4 = severe). Dummy variables were created for all of the independent variables in the modeling. The statistical analyses were carried out using the STATA 16 SE version. Statistical significance was set at 0.05 using two-sided tests.

## Result

### Characteristics of respondents

From February 2021 to November 2022, a total of 7,604 respondents were involved, of which 106 interviews were excluded because the respondents did not complete the whole interview (*N* = 67), or the interviews did not meet the inclusion criteria (*N* = 5), or answered with logical inconsistencies (*N* = 17), or the interview took less than 5 min (*N* = 17). Finally, a total of 7,498 respondents were included.

The sample relative approximate to the general adult Chinese population in terms of age, sex, ethnicity, and district of residence, each geographic distribution ranged from 9.71 to 25.54% (compared with Communiqué of the Seventh National Population Census and China Statistical Yearbook of 2023 in Table 1, and see Table 2 for details). Of the respondents, 45.84% were men and 54.16% were women. About 89.82% of the respondents were Han ethnicity. And 62.34% of respondents were married and 32.24% were unmarried. 52.28% of respondents were rural householding registration. About 20% of the respondents earn less than 1,300 CNY per month. A considerable proportion of the population possesses a higher level of education, with nearly 50% holding a university degree, while approximately 10% have completed only primary-level education. 50.12% of respondents were employed and 8.58% were unemployed. About 40% of respondents reported the presence of chronic conditions. More than 70% of respondents reported participating in physical activities frequently or occasionally. 74.58% of respondents were non-smokers and 53.77% were non-drinkers (Table 2).

**Table 1:** Compare the distribution of sociodemographic characteristics with the Chinese census

Characteristics	Chinese general population ^a.b^ (%)	Sample in this research (%)
**Sex** ^**a**^		
Male	51.24	45.84
Female	48.76	54.16
**Ethnic** ^**a**^		
Han nationality	91.11	89.82
Ethnic minorities	8.89	10.18
**Geographical division** ^**b**^		
East China	30.20	25.54
Central and South China	29.12	26.97
Northeast China	6.84	9.71
North China	11.95	12.52
Northwest China	7.34	10.26
Southwest China	14.54	15.00
**Education level** ^**a**^		
University	15.13	48.88
High school/Junior college	14.76	24.43
Middle school	33.75	16.66
Primary school and below	26.84	10.03
**Age** ^**a**^		
15—59 years	82.43	Almost 81.18
60 + years	17.57	Almost 18.82
**Age** ^**b**^		
15∼24 years	12.77	25.47
25∼34 years	16.83	14.94
35∼44 years	17.36	14.90
45∼54 years	19.31	20.10
55∼64 years	15.80	11.55
65∼74 years	11.57	7.00
75 + years	6.36	6.04
**Household registration** ^**a**^		
Non-rural	63.89	57.00
Rural	36.11	43.00

*Note*^a^ the data is sourced from the Seventh National Population Census of China in 2020 https://www.stats.gov.cn/sj/zxfb/202302/t20230203_1901085.html;^b^ the data is sourced from the China Statistical Yearbook of 2023 https://www.stats.gov.cn/sj/ndsj/2023/indexch.htm

**Table 2:** CQ-11D index scores and demographic characteristics of all respondents

Characteristics	N (%)	Mean(sd)	Q1 ∼ Q3	K-W/Wilcoxon
Total	7498(100.00)	0.897(0.142)	0.870 ∼ 0.983	-
**Sex**				**< 0.001**
Male	3437(45.84)	0.910(0.131)	0.891 ∼ 0.989	
Female	4061(54.16)	0.886(0.150)	0.863 ∼ 0.976	
**Age**				**< 0.001**
15∼24 years	1910(25.47)	0.921(0.107)	0.902 ∼ 0.989	
25∼34 years	1120(14.94)	0.921(0.118)	0.909 ∼ 0.994	
35∼44 years	1117(14.90)	0.915(0.111)	0.893 ∼ 0.987	
45∼54 years	1507(20.10)	0.899(0.123)	0.873 ∼ 0.978	
55∼64 years	866(11.55)	0.879(0.155)	0.857 ∼ 0.972	
65∼74 years	525(7.00)	0.835(0.194)	0.784 ∼ 0.957	
75 + years	453(6.04)	0.796(0.248)	0.731 ∼ 0.964	
**Geographical division**				**< 0.001**
North China	939(12.52)	0.894(0.121)	0.861 ∼ 0.967	
Central China	1129(15.06)	0.884(0.148)	0.848 ∼ 0.983	
East China	1915(25.54)	0.899(0.152)	0.878 ∼ 0.983	
South China	893(11.91)	0.898(0.142)	0.868 ∼ 0.994	
Northeast China	728(9.71)	0.904(0.146)	0.883 ∼ 0.989	
Northwest China	769(10.26)	0.897(0.133)	0.869 ∼ 0.976	
Southwest China	1125(15.00)	0.905(0.137)	0.884 ∼ 0.987	
**Ethnicity**				**0.772**
Han nationality	6735(89.82)	0.899(0.139)	0.874 ∼ 0.983	
Ethnic minorities	763(10.18)	0.881(0.167)	0.841 ∼ 0.994	
**Marital status**				**< 0.001**
Unmarried	2567(34.23)	0.918(0.115)	0.897 ∼ 0.993	
Married	4674(62.34)	0.891(0.146)	0.867 ∼ 0.978	
Divorced/widowed	246(3.28)	0.797(0.243)	0.716 ∼ 0.961	
Others	11(0.15)	0.881(0.108)	0.816 ∼ 0.958	
**Occupation**				**< 0.001**
Employed	3758(50.12)	0.909(0.121)	0.882 ∼ 0.983	
Retirement	910(12.14)	0.853(0.176)	0.814 ∼ 0.961	
Student	1989(26.53)	0.915(0.123)	0.898 ∼ 0.989	
Unemployed	643(8.57)	0.832(0.216)	0.781 ∼ 0.967	
Others	198(2.64)	0.917(0.109)	0.902 ∼ 0.989	
**Household registration**				**0.8724**
Non-rural	4274(57)	0.904(0.125)	0.875 ∼ 0.982	
Rural	3224(43)	0.888(0.162)	0.867 ∼ 0.989	
**Education level**				**< 0.001**
Primary school and below	752(10.03)	0.825(0.210)	0.770 ∼ 0.964	
Middle school	1249(16.66)	0.892(0.150)	0.869 ∼ 0.983	
High school/Junior college	1832(24.43)	0.899(0.139)	0.874 ∼ 0.989	
University	3235(43.15)	0.915(0.114)	0.891 ∼ 0.987	
Master’s degree and above	430(5.73)	0.899(0.133)	0.870 ∼ 0.978	
**Income/month, RMB**				**0.061**
0 ∼ 1300	1636(21.82)	0.883(0.168)	0.858 ∼ 0.987	
1300–3300	2264(30.20)	0.901(0.133)	0.873 ∼ 0.983	
3300–6300	2090(27.87)	0.901(0.132)	0.875 ∼ 0.982	
6300–13,000	1061(14.15)	0.907(0.127)	0.881 ∼ 0.987	
13,000–21,000	219(2.92)	0.893(0.131)	0.867 ∼ 0.971	
21,000–42,000	84(1.12)	0.898(0.143)	0.874 ∼ 1.000	
42,000 and above	144(1.92)	0.887(0.190)	0.867 ∼ 0.997	
**Smoking**				**0.004**
Never smoked	5592(74.58)	0.904(0.130)	0.875 ∼ 0.983	
Occasional smoker	596(7.95)	0.880(0.163)	0.846 ∼ 0.982	
Frequent smoker	1020(13.60)	0.878(0.174)	0.863 ∼ 0.983	
Former smoker	290(3.87)	0.875(0.182)	0.864 ∼ 0.978	
**Drinking**				**< 0.001**
Never drink	4032(53.77)	0.901(0.140)	0.876 ∼ 0.989	
Occasional drinker	2550(34.01)	0.908(0.120)	0.878 ∼ 0.982	
Frequent drinker	566(7.55)	0.842(0.204)	0.790 ∼ 0.967	
Former drinker	350(4.67)	0.866(0.167)	0.835 ∼ 0.967	
**Participation in physical activities**				**< 0.001**
Frequent participation	2621(34.96)	0.913(0.128)	0.897 ∼ 0.994	
Occasional participation	2958(39.45)	0.906(0.123)	0.878 ∼ 0.978	
Never participate	1669(22.26)	0.861(0.179)	0.818 ∼ 0.972	
Uncertain	250(3.33)	0.878(0.165)	0.837 ∼ 0.987	
**Changes in self-perceived health status compared to the previous year**				**< 0.001**
No change	3222(42.97)	0.930(0.097)	0.918 ∼ 0.993	
Improved	1865(24.87)	0.913(0.130)	0.895 ∼ 0.994	
Worsened	1351(18.02)	0.811(0.201)	0.748 ∼ 0.942	
Uncertain	1060(14.14)	0.882(0.139)	0.857 ∼ 0.965	
**Presence of chronic diseases**				**< 0.001**
No	4520(60.28)	0.933(0.090)	0.918 ∼ 0.994	
Yes	2978(39.72)	0.843(0.184)	0.801 ∼ 0.960	
Category of chronic diseases^a^				-
-Cardiovascular disease^b^	238(3.17)	0.772(0.227)	0.688 ∼ 0.931	
-Hypertension	783(10.44)	0.825(0.205)	0.775 ∼ 0.953	
-Stroke or other cerebrovascular diseases	71(0.95)	0.725(0.265)	0.574 ∼ 0.924	
-Diabetes	236(3.15)	0.814(0.197)	0.731 ∼ 0.958	
-Chronic respiratory disease^c^	137(1.83)	0.819(0.200)	0.727 ∼ 0.947	
-Arthritis^d^	483(6.44)	0.803(0.211)	0.742 ∼ 0.931	
-Osteoporosis or primary osteoporosis	105(1.40)	0.750(0.265)	0.665 ∼ 0.929	
-Cancer or malignant tumor	67(0.89)	0.750(0.259)	0.608 ∼ 0.965	
-Other chronic diseases	1379(18.39)	0.864(0.162)	0.828 ∼ 0.964	
**Number of chronic diseases**				**0.003**
1	2584(34.46)	0.858(0.168)	0.821 ∼ 0.961	
2	292(3.89)	0.764(0.235)	0.681 ∼ 0.925	
3	80(1.07)	0.728(0.240)	0.650 ∼ 0.905	
4	20(0.27)	0.612(0.319)	0.469 ∼ 0.861	

*Note*: ^a^ When calculating the proportion of chronic diseases in each category, the denominator is the entire population; ^b^ Such as myocardial infarction, coronary heart disease, congestive heart failure, and other cardiac diseases; ^c^ Such as chronic bronchitis or emphysema; ^d^ Rheumatoid arthritis, osteoarthritis, gouty arthritis

### Primary outcomes

The mean CQ-11D index scores were 0.897(SD: 0.142). The mean CQ-11D index scores of presence of chronic disease respondents were 0.843(SD: 0.184): 0.772(SD: 0.227) for cardiovascular disease, 0.825(SD: 0.205) for hypertension, 0.725(SD: 0.265) for stroke or other cerebrovascular diseases, 0.814(SD: 0.197) for diabetes, 0.819(SD: 0.200) for chronic respiratory disease, 0.803(SD: 0.211) for arthritis, 0.750(SD: 0.265) for osteoporosis or primary osteoporosis, and 0.750 (SD: 0.259) cancer or malignant tumor (Table 2). The mean utility score ranged from 0.921 ± 0.107 (age group 16 ∼ 24) to 0.796 ± 0.248 (age group 75+). Female respondents had lower CQ-11D scores (Mean 0.886, SD 0.150) than male respondents (Mean 0.910, SD 0.131) with the p-values < 0.001. The CQ-11D index scores varied with the demographic and socioeconomic characteristics of respondents, except for ethnicity (*p* > 0.05) and income (*p* > 0.05). The lower CQ-11D index scores were associated with older age, being female, being married or widowed, unemployment (including being retired), rural household registration, smoking, drinking, not exercising, lower income, and chronic disease conditions. Respondents in the Northeast region had the highest CQ-11D index scores among the seven geographic divisions. Respondents with primary level education or below had lower CQ-11D index scores (0.825) and those with a university degree education had higher CQ-11D index scores (0.915).

**Fig. 1:**
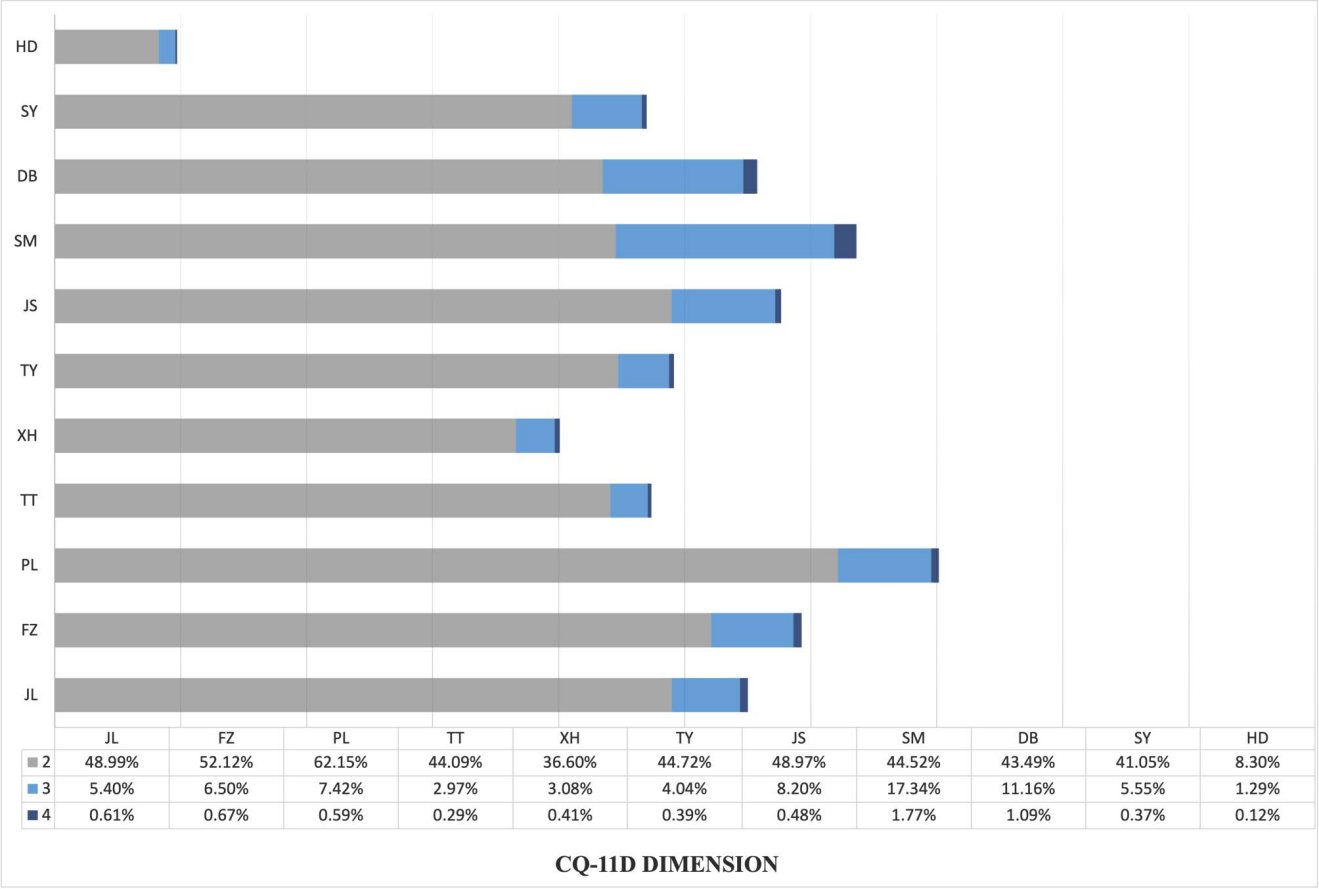
Frequencies of having “any problems” (level 2–4) in the CQ-11D dimensions in the whole sample

**Fig. 2:**
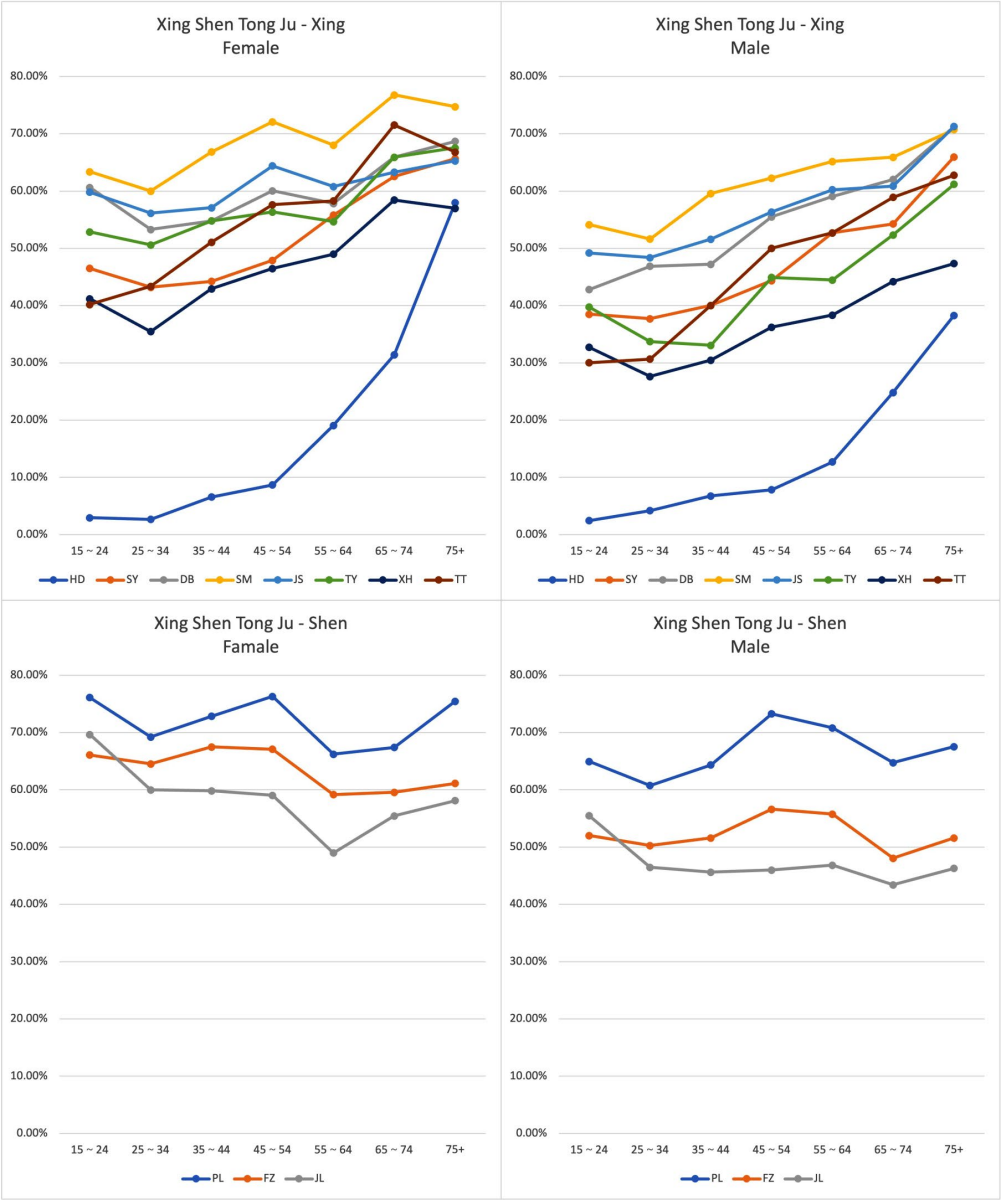
Frequencies of having “any problems” (level 2–4) in the CQ-11D dimensions presented by sex and age groups

In total, according to the responses of the individual CQ-11D dimensions, most health problems were identified in the PL (70.16%) and SM (63.63%) dimensions (Fig. 1). The percentage of “non-problem” were: 90.29% for XD, 53.03% for SY, 44.25% for DB, 36.37% for SM, 42.34% for JS, 50.85% for TY, 59.91% for XH, 52.64% for TT, 29.85% for PL, 40.72% for FZ, and 45.00% for JL. The percentage of reported problems for each level CQ-11D dimension for sex and age groups (Tables 3 and 4, and Fig. 2). The XING dimensions (XD, SM, DB, TY, SY, JS, TT, and XH) were relatively low percentages of any health problem in the younger age group, which increased with increasing age. The percentage of SHEN dimensions (PL, FZ, and JL) that reported any problems remained at a high level of about 45%∼75% across all age groups. The percentage of respondents who reported any problems in the PL dimension was higher in the 15 ∼ 24, 45 ∼ 54, and 75 + age groups. The proportion of participants who indicated difficulties in the FZ dimension and JL dimension was considerable, with a consistently high level of constraints (> 60%), which remained relatively steady across various age cohorts. We found significant differences between male and female respondents in every health dimension, except for the XD dimension (Table 3). For male respondents, there was a sharp increase in the age groups of 65 to 75 + for all health dimensions. This sharp increase was observed among female respondents in the age groups of 55 ∼ 64 to 65 ∼ 74. Once female respondents reached 75 + years old, the percentage of any problem in dimensions of SM, TT, and XH decreased. In general, a higher percentage of female respondents than males reported any problem across all dimensions.

**Table 3:** Percentage of reported problems in 11 health dimensions of CQ-11D presented by sex and age group

CQ-11D dimension		Male (%)	Female (%)	All (%)	X^2^	15∼24 (%)	25∼34 (%)	35∼44 (%)	45∼54 (%)	55∼64 (%)	65∼74 (%)	75+ (%)	X^2^
HD	Level 1	90.60	90.03	90.29	3.0789	97.70	96.78	94.00	92.04	84.41	72.38	60.05	Fisher**
	Level 2	8.23	8.35	8.30		1.73	2.59	5.46	7.50	14.20	23.81	30.46	
	Level 3	1.05	1.50	1.29		0.52	0.63	0.36	0.46	1.27	3.81	8.39	
	Level 4	0.12	0.12	0.12		0.05	0.00	0.18	0.00	0.12	0.00	1.10	
	Any problem (level 2–4)	9.40	9.97	9.71		2.30	3.22	6.00	7.96	15.59	27.62	39.95	
SY	Level 1	55.72	50.76	53.03	24.6496**	56.91	59.38	57.65	53.81	45.73	41.52	34.22	186.2923**
	Level 2	39.39	42.45	41.05		37.70	37.23	37.96	40.88	47.00	47.43	54.08	
	Level 3	4.54	6.40	5.55		4.92	3.21	4.21	4.98	6.81	10.29	11.26	
	Level 4	0.35	0.39	0.37		0.47	0.18	0.18	0.33	0.46	0.76	0.44	
	Any problem (level 2–4)	44.28	49.24	46.97		43.09	40.62	42.35	46.19	54.27	58.48	65.78	
DB	Level 1	48.25	40.88	44.26	58.7996**	46.96	49.73	48.61	42.13	41.57	36.00	30.25	132.807**
	Level 2	42.01	44.74	43.49		43.04	39.29	40.11	46.32	46.19	46.10	46.58	
	Level 3	8.93	13.05	11.16		9.06	10.09	10.12	10.62	10.97	16.00	21.85	
	Level 4	0.81	1.33	1.09		0.94	0.89	1.16	0.93	1.27	1.90	1.32	
	Any problem (level 2–4)	51.75	59.12	55.74		53.04	50.27	51.39	57.87	58.43	64.00	69.75	
SM	Level 1	40.59	32.80	36.37	60.1938**	40.58	43.93	36.44	32.64	33.37	28.57	26.93	142.6997**
	Level 2	42.89	45.90	44.52		43.98	40.18	47.54	45.99	44.11	44.76	45.70	
	Level 3	15.27	19.08	17.34		14.03	14.55	15.04	19.91	19.75	23.62	23.40	
	Level 4	1.25	2.22	1.77		1.41	1.34	0.98	1.46	2.77	3.05	3.97	
	Any problem (level 2–4)	59.41	67.20	63.63		59.42	56.07	63.56	67.36	66.63	71.43	73.07	
JS	Level 1	45.62	39.57	42.35	33.8023**	44.71	47.50	45.39	39.48	39.49	37.91	32.23	117.516**
	Level 2	47.02	50.63	48.97		48.01	44.82	48.88	52.36	49.77	49.14	50.55	
	Level 3	6.95	9.26	8.20		7.07	7.50	5.46	7.50	10.16	12.00	15.67	
	Level 4	0.41	0.54	0.48		0.21	0.18	0.27	0.66	0.58	0.95	1.55	
	Any problem (level 2–4)	54.38	60.43	57.65		55.29	52.50	54.61	60.52	60.51	62.09	67.77	
TY	Level 1	58.36	44.50	50.85	155.1893**	52.72	57.32	54.97	49.16	50.34	40.77	35.10	172.9124**
	Level 2	38.70	49.82	44.72		44.71	39.46	41.99	46.65	43.42	49.71	54.75	
	Level 3	2.59	5.27	4.04		2.36	2.59	2.86	3.92	5.66	8.57	9.71	
	Level 4	0.35	0.42	0.39		0.21	0.63	0.18	0.27	0.58	0.95	0.44	
	Any problem (level 2–4)	41.64	55.50	49.15		47.28	42.68	45.03	50.84	49.66	59.23	64.90	
XH	Level 1	65.29	55.36	59.91	79.4047**	62.41	68.21	62.67	58.46	56.24	48.57	47.03	172.1743**
	Level 2	32.06	40.43	36.60		35.92	29.55	34.91	38.02	38.45	42.86	45.47	
	Level 3	2.36	3.69	3.08		1.57	1.88	2.15	3.12	4.50	7.62	6.62	
	Level 4	0.29	0.52	0.41		0.10	0.36	0.27	0.40	0.81	0.95	0.88	
	Any problem (level 2–4)	34.71	44.64	40.09		37.59	31.79	37.33	41.54	43.76	51.43	52.97	
TT	Level 1	57.43	48.58	52.65	58.7932**	64.13	62.59	53.90	46.05	44.46	34.67	34.88	Fisher**
	Level 2	39.69	47.82	44.09		34.87	35.89	44.58	50.83	50.35	55.81	54.08	
	Level 3	2.59	3.30	2.97		0.84	1.25	1.43	2.92	4.73	8.38	10.60	
	Level 4	0.29	0.30	0.29		0.16	0.27	0.09	0.20	0.46	1.14	0.44	
	Any problem (level 2–4)	42.57	51.42	47.35		35.87	37.41	46.10	53.95	55.54	65.33	65.12	
PL	Level 1	33.17	27.04	29.84	34.296**	28.65	34.73	30.97	25.15	31.52	33.90	27.81	98.411**
	Level 2	59.27	64.59	62.15		64.76	58.57	62.13	67.15	59.82	54.48	56.73	
	Level 3	6.92	7.83	7.42		6.07	6.34	6.45	7.30	7.97	10.67	13.69	
	Level 4	0.64	0.54	0.59		0.52	0.36	0.45	0.40	0.69	0.95	1.77	
	Any problem (level 2–4)	66.83	72.96	70.16		71.35	65.27	69.03	74.85	68.48	66.10	72.19	
FZ	Level 1	47.19	35.24	40.71	120.0038**	39.89	42.14	39.66	37.96	42.49	46.10	42.83	51.4314**
	Level 2	47.25	56.24	52.12		54.66	51.16	51.92	54.28	49.08	46.29	49.67	
	Level 3	5.21	7.58	6.50		5.08	6.07	7.97	7.03	7.97	5.71	6.18	
	Level 4	0.35	0.94	0.67		0.37	0.63	0.45	0.73	0.46	1.90	1.32	
	Any problem (level 2–4)	52.81	64.76	59.29		60.11	57.86	60.34	62.04	57.51	53.90	57.17	
JL	Level 1	51.82	39.23	45.00	122.4692**	36.39	46.34	46.55	47.24	52.07	50.48	46.81	104.1665**
	Level 2	43.26	53.83	48.99		56.54	48.48	49.06	46.72	42.96	41.90	45.47	
	Level 3	4.54	6.13	5.40		6.34	4.38	4.12	5.44	4.62	7.05	6.62	
	Level 4	0.38	0.81	0.61		0.73	0.80	0.27	0.60	0.35	0.57	1.10	
	Any problem (level 2–4)	48.18	60.77	55.00		63.61	53.66	53.45	52.76	47.93	49.52	53.19	

*Note* ** indicates *P* < 0.001

**Table 4:** Percentage of reported any problems in 11 health dimensions of CQ-11D presented by sex and age group

Dimension	Male								Female						
	15∼24 (%)	25∼34 (%)	35∼44 (%)	45∼54 (%)	55∼64 (%)	65∼74 (%)	75+ (%)	15∼24 (%)		25∼34 (%)	35∼44 (%)	45∼54 (%)	55∼64 (%)	65∼74 (%)	75+ (%)
XD	2.46	4.19	6.77	7.85	12.71	24.81	38.30	2.95		2.67	6.57	8.68	19.06	31.40	57.98
SY	38.50	37.71	40.04	44.35	52.71	54.26	65.96	46.49		43.19	44.23	47.89	55.78	62.55	65.66
DB	42.80	46.86	47.21	55.51	59.06	62.02	71.28	60.62		53.28	54.80	60.05	57.82	65.92	68.68
SM	54.12	51.62	59.56	62.26	65.18	65.89	70.74	63.35		60.00	66.83	72.09	68.03	76.78	74.72
JS	49.20	48.38	51.59	56.34	60.24	60.85	71.28	59.80		56.13	57.07	64.40	60.77	63.30	65.28
TY	39.73	33.71	33.07	44.90	44.47	52.33	61.17	52.87		50.59	54.80	56.34	54.65	65.92	67.55
XH	32.72	27.62	30.48	36.23	38.35	44.19	47.34	41.20		35.46	42.93	46.48	48.98	58.43	56.98
TT	30.01	30.67	40.04	50.00	52.71	58.91	62.77	40.20		43.36	51.06	57.62	58.28	71.54	66.79
PL	64.94	60.76	64.34	73.28	70.82	64.73	67.55	76.12		69.24	72.85	76.31	66.21	67.42	75.47
FZ	52.03	50.29	51.59	56.61	55.76	48.06	51.60	66.09		64.54	67.48	67.09	59.18	59.55	61.13
JL	55.47	46.48	45.62	46.01	46.82	43.41	46.28	69.64		60.00	59.84	59.03	48.98	55.43	58.11

#### Multivariable regression

Table 5 shows the results of multivariate analysis on socio-demographic characteristics and health-relative variables. The sex (female), older than 65 age, ethnicity of non-Han, being widowed or divorced, primary education level or below, household registration (rural), students or unemployed, smoking (occasionally, frequent, or former smoker), drinking (occasionally, frequent, or former drinker), physical activity(occasionally, never, or uncertain), changes self-perceived health status compared to the previous year (improved, worsened, or uncertain), and presence of chronic diseases were negative association with health utility scores and both significant. The North, Central, and West-north geographic divisions, rural household registration, retirement, and being married were negative but not significantly associated with HRQoL. Compared with a monthly income of less than 1300 CNY, when the monthly income increased to 1300 ∼ 13,000 CNY, there was a significant positive correlation with the health utility value.

**Table 5:** Associations between characteristics and CQ-11D index scores

	Coef.	t	P	Beta	Likelihood’ chi-squared	P
Sex					*123.71*	< 0.001
Male	Ref.					
Female	-0.039	-11.122	< 0.001	-0.137		
Age					*15.28*	< 0.001
15 ∼ 24	Ref.					
25∼34	0.002	0.349	0.730	0.006		
35∼44	0.004	0.487	0.630	0.009		
45∼54	-0.003	-0.392	0.690	-0.008		
55∼64	-0.011	-1.339	0.180	-0.025		
65∼74	-0.031	-3.377	< 0.001	-0.056		
75 + years	-0.065	-7.328	< 0.001	-0.108		
Ethnicity					8.26	0.004
Han nationality	Ref.					
Ethnic minorities	-0.014	-2.873	< 0.001	-0.030		
Geographical division					2.96	0.007
East China	Ref.					
North China	-0.009	-1.805	0.070	-0.021		
Central China	-0.009	-1.904	0.060	-0.023		
South China	0.003	0.669	0.500	0.008		
Northeast China	0.005	0.854	0.390	0.010		
Northwest China	-0.004	-0.770	0.440	-0.009		
Southwest China	0.008	1.747	0.080	0.021		
Marital status					15.67	< 0.001
Unmarried	Ref.					
Married	-0.008	-1.379	0.170	-0.029		
Divorced/widowed	-0.064	-6.300	< 0.001	-0.080		
Others	-0.019	-0.493	0.620	-0.005		
Education level					5.25	< 0.001
Master's degree and above	Ref.					
Primary school and below	-0.022	-2.548	0.010	-0.047		
Middle school	0.002	0.321	0.750	0.006		
High school/Junior college	0.003	0.376	0.710	0.008		
University	0.005	0.789	0.430	0.018		
Household registration					5.66	0.017
Non-rural	Ref.					
Rural	-0.008	-2.379	0.020	-0.028		
Occupation					8.27	< 0.001
Employed	Ref.					
Retirement	-0.003	-0.546	0.590	-0.007		
Student	-0.021	-3.012	< 0.001	-0.066		
Unemployed	-0.023	-3.754	< 0.001	-0.045		
Others	0.028	3.063	< 0.001	0.032		
Income/month, RMB					3.38	0.003
0 ∼ 1300	Ref.					
1300–3300	0.017	3.847	< 0.001	0.053		
3300–6300	0.010	1.855	0.060	0.030		
6300–13000	0.014	2.261	0.020	0.034		
13000–21000	-0.003	-0.293	0.770	-0.003		
21000–42000	0.002	0.156	0.880	0.002		
42000 and above	-0.004	-0.342	0.730	-0.004		
Smoking					11.66	< 0.001
Never smoked	Ref.					
Occasional smoker	-0.030	-5.079	< 0.001	-0.057		
Frequent smoker	-0.023	-4.382	< 0.001	-0.055		
Former smoker	-0.016	-1.952	0.050	-0.022		
Drinking					18.37	< 0.001
Never drink	Ref.					
Occasional drinker	-0.007	-2.091	0.040	-0.024		
Frequent drinker	-0.046	-7.160	< 0.001	-0.086		
Former drinker	-0.022	-2.933	< 0.001	-0.032		
Participation in physical activities					23.63	< 0.001
Frequent participation	Ref.					
Occasional participation	-0.010	-3.005	< 0.001	-0.036		
Never participate	-0.034	-8.292	< 0.001	-0.099		
Uncertain	-0.022	-2.582	0.010	-0.027		
Changes in self-perceived health status compared to the previous year					161.30	< 0.001
No change	Ref.					
Improved	-0.018	-4.853	< 0.001	-0.054		
Worsened	-0.090	-21.686	< 0.001	-0.244		
Uncertain	-0.039	-8.582	< 0.001	-0.095		
Presence of chronic diseases					389.89	< 0.001
No	Ref.					
Yes	-0.065	-19.746	< 0.001	-0.223		

### Ordinal logistic regression

Table 6 shows the results of ordinal logistic regression on socio-demographic characteristics and health-relative variables. The female respondents had significantly higher odds of reported problems in all of the 11 dimensions. Compared with the 15 ∼ 24 age group, older age groups had lower odds of reporting health problems of SHEN dimensions (OR 0.49 ∼ 0.76). The odds of reporting problems with XD dimension increase with age (OR 3.16 ∼ 10.17). After the age of 65 years, the odds of reporting problems in the XING dimensions (SY, DB, TY, XH, and TT) are significantly increased (OR 1.38 ∼ 1.73). Compared with ethnic Han respondents, minority ethic respondents had higher odds of reporting problems were higher in XD and SY dimensions (OR 1.21 ∼ 1.90) and lower in the PL and FZ dimensions (OR 0.79 ∼ 0.80). Respondents who had experienced marriage had higher odds of reporting problems both in the XING and SHEN dimensions, especially those who were divorced and widowed respondents (OR 1.26 ∼ 2.13). The odds of reporting health problems increased with educational attainment in 3 dimensions of SHEN dimensions (PL, FZ, and JL), with those who were High school/Junior college and university educated having an OR 1.25  1.35 compared with those with higher than primary school education. Compared with employed respondents, unemployed respondents had higher odds of reporting health problems in some dimensions of XING dimensions (XD, SY, DB, JS, and TY) and had lower odds of reporting health problems in PL. Retired respondents had higher odds of reporting health problems in some dimensions of XING dimensions (XD, DB, SM, and TT), which may be related to the older age of retired respondents compared with employed respondents.

Among health indicators, occasionally/often smoking (OR 1.5524 ∼ 2.8399), occasionally/often drinking (OR 1.14 ∼ 2.8307), occasionally/never participated in physical activities (OR 1.25 ∼ 2.13), and worsened/uncertain changes in self-perceived health status compared to the previous year (OR 1.47 ∼ 3.49) increase the odds of reporting problems in almost all of the dimensions. Compared to non-smokers, occasional smokers have some negative impact on XD, SY, DB, JS, TY, XH, TT, and PL dimensions, with an OR range of 1.23 to 2.83. Frequent smokers compared to non-smokers have negative impacts on XD, DB, XH, PL, and FZ dimensions, with an OR range of 1.20 to 1.55. Former smokers compared to non-smokers have negative impacts on the XH dimension, with an OR of 1.35. It can be seen that the number of dimensions with negative impacts is former smokers < frequent smokers < occasional smokers. For drinkers, there were similar results. Compared to non-drinkers, occasional drinkers have some negative effect on DB, SM, JS, TY, XH, TT, PL, FZ, and JL dimensions, with an OR range of 1.14 to 1.43; Frequent drinkers have some negative effect on XD, SY, DB, SM, TY, XH, TT, PL, FZ and JL, with OR range of 1.29 to 2.11; former drinkers have some negative effect on DB, SM, JS, TT, PL and JL, with an OR range of 1.33 to 1.68. It can be seen that the number of dimensions with negative impacts is former drinkers < occasional drinkers < frequent drinkers. Respondents with chronic conditions had an OR 1.62 ∼ 4.23 for reporting problems across all dimensions, especially the XD dimension (OR = 4.23).

**Table 6:** Associations between characteristics and health problems reported in 11 dimensions

	XD OR(95%CI)	SY OR(95%CI)	DB OR(95%CI)	SM OR(95%CI)	JS OR(95%CI)	TY OR(95%CI)	XH OR(95%CI)	TT OR(95%CI)	PL OR(95%CI)	FZ OR(95%CI)	JL OR(95%CI)
Sex											
Male	Ref.	Ref.	Ref.	Ref.	Ref.	Ref.	Ref.	Ref.	Ref.	Ref.	Ref.
Female	1.72(1.36,2.18)**	1.37(1.22,1.53)**	1.63(1.46,1.82)**	1.53(1.38,1.70)**	1. 45(1.30,1.63)**	2.13(1.90,2.40)**	1.86(1.65,2.11)**	1.94(1.72,2.20)**	1.64(1.45,1.85)**	2.00(1.79,2.24)**	1.87(1.66,2.09)**
**Age**											
15 ∼ 24	Ref.	Ref.	Ref.	Ref.	Ref.	Ref.	Ref.	Ref.	Ref.	Ref.	Ref.
25∼34	1.00(0.51,1.96)	0.84(0.67,1.05)	0.91(0.74,1.13)	0.87(0.70,1.08)	0.84(0.67,1.04)	0.99(0.80,1.23)	0.87(0.69,1.10)	0.80(0.63,1.01)	0.57(0.46,0.72)**	0.76(0.61,0.93)*	0.74(0.59,0.92)*
35∼44	1.66(0.84,3.29)	0.87(0.68,1.13)	0.93(0.73,1.19)	1.01(0.79,1.30)	0.80(0.62,1.03)	1.06(0.83,1.36)	1.14(0.87,1.49)	0.95(0.73,1.25)	0.57(0.44,0.74)**	0.79(0.62,1.01)	0.74(0.57,0.95)*
45∼54	1.87(0.99,3.55)	0.98(0.76,1.26)	1.09(0.86,1.38)	1.19(0.93,1.51)	0.99(0.78,1.27)	1.25(0.97,1.59)	1.24(0.95,1.61)	1.18(0.91,1.53)	0.66(0.51,0.86)*	0.79(0.62,1.01)	0.73(0.57,0.93)*
55∼64	3.16(1.69,5.93)**	1.24(0.95,1.62)	1.01(0.78,1.30)	1.09(0.84,1.42)	0.98(0.75,1.29)	1.14(0.87,1.50)	1.31(0.98,1.73)	1.15(0.87,1.53)	0.55(0.41,0.73)**	0.69(0.53,0.90)*	0.62(0.47,0.81)*
65∼74	5.21(2.76,9.81)**	1.38(1.02,1.86)*	1.17(0.87,1.56)	1.20(0.89,1.61)	0.94(0.70,1.28)	1.56(1.15,2.12)*	1.59(1.15,2.18)*	1.55(1.12,2.14)*	0.49(0.35,0.68)**	0.57(0.42,0.77)**	0.64(0.47,0.87)*
≥ 75	10.17(5.68,18.20)**	1.70(1.28,2.25)**	1.45(1.10,1.91)*	1.23(0.92,1.64)	1.18(0.87,1.59)	1.73(1.30,2.30)**	1.46(1.09,1.96)*	1.64(1.20,2.22)*	0.70(0.51,0.95)*	0.59(0.44,0.78)**	0.61(0.45,0.83)*
**Ethnicity**											
Han nationality	Ref.	Ref.	Ref.	Ref.	Ref.	Ref.	Ref.	Ref.	Ref.	Ref.	Ref.
Ethnic minorities	1.90(1.47,2.45)**	1.21(1.03,1.41)*	1.15(0.99,1.34)	0.96(0.83,1.12)	1.06(0.90,1.24)	1.09(0.93,1.28)	1.04(0.88,1.23)	0.94(0.79,1.12)	0.80(0.68,0.95)*	0.79(0.67,0.93)*	0.91(0.77,1.07)
**Geographical division**											
East China	Ref.	Ref.	Ref.	Ref.	Ref.	Ref.	Ref.	Ref.	Ref.	Ref.	Ref.
North China	1.07(0.78,1.48)	1.00(0.85,1.18)	1.20(1.02,1.40)*	1.24(1.07,1.43)*	1.14(0.98,1.34)	1.27(1.08,1.49)*	1.26(1.06,1.50)*	1.33(1.13,1.58)*	1.23(1.05,1.44)*	0.90(0.77,1.06)	1.06(0.91,1.24)
Central China	1.30(0.99,1.70)	1.16(0.99,1.35)	1.15(1.00,1.34)	1.05(0.91,1.21)	1.09(0.94,1.26)	1.17(1.00,1.36)*	1.13(0.96,1.32)	1.21(1.03,1.41)*	0.97(0.83,1.13)	1.23(1.06,1.43)*	1.12(0.96,1.30)
South China	1.02(0.76,1.37)	0.97(0.82,1.13)	0.81(0.69,0.95)*	0.87(0.74,1.01)	0.92(0.78,1.08)	1.08(0.91,1.27)	1.23(1.04,1.46)*	1.01(0.85,1.20)	1.04(0.88,1.23)	1.04(0.88,1.22)	1.06(0.90,1.25)
Northeast China	0.85(0.59,1.22)	0.89(0.74,1.06)	1.03(0.87,1.21)	0.86(0.73,1.01)	0.68(0.57,0.82)**	0.88(0.73,1.06)	1.19(0.98,1.44)	0.86(0.71,1.04)	0.84(0.70,1.01)	0.85(0.71,1.01)	0.73(0.61,0.87)*
Northwest China	0.80(0.57,1.14)	0.97(0.82,1.15)	1.34(1.13,1.57)*	1.05(0.90,1.23)	1.13(0.96,1.34)	1.13(0.94,1.34)	1.27(1.06,1.52)*	1.18(0.98,1.42)	1.28(1.07,1.53)*	1.10(0.93,1.30)	1.34(1.13,1.59)*
Southwest China	0.99(0.73,1.34)	0.98(0.84,1.15)	0.84(0.73,0.98)*	0.81(0.69,0.94)*	0.73(0.62,0.85)**	0.95(0.80,1.11)	1.04(0.87,1.23)	0.88(0.74,1.04)	0.69(0.59,0.81)**	0.91(0.78,1.07)	0.85(0.73,0.99)*
**Marital status**											
Unmarried	Ref.	Ref.	Ref.	Ref.	Ref.	Ref.	Ref.	Ref.	Ref.	Ref.	Ref.
Married	1.16(0.68,1.99)	1.01(0.82,1.23)	1.01(0.84,1.22)	1.16(0.96,1.40)	1.20(0.98,1.47)	1.05(0.86,1.28)	0.94(0.76,1.16)	1.31(1.07,1.62)*	1.32(1.08,1.62)*	1.26(1.04,1.53)*	1.05(0.87,1.28)
Divorced/widowed	1.92(1.03,3.57)*	1.44(1.02,2.02)*	1.42(1.03,1.96)*	1.60(1.17,2.20)*	1.99(1.41,2.81)**	1.20(0.87,1.65)	1.32(0.95,1.85)	1.59(1.12,2.25)*	2.13(1.51,3.02)**	1.47(1.07,2.02)*	1.43(1.03,2.01)*
Others	0.00(0.00,0.00)**	1.92(0.37,10.09)	1.71(0.31,9.51)	2.07(1.02,4.19)*	1.62(0.59,4.46)	0.73(0.19,2.74)	1.29(0.38,4.35)	2.82(0.60,13.19)	0.73(0.20,2.61)	1.53(0.43,5.46)	1.85(0.68,5.02)
**Education level**											
Primary school and below	Ref.	Ref.	Ref.	Ref.	Ref.	Ref.	Ref.	Ref.	Ref.	Ref.	Ref.
Middle school	0.74(0.56,0.98)*	0.98(0.81,1.19)	0.88(0.73,1.06)	1.01(0.83,1.23)	0.87(0.71,1.06)	0.91(0.73,1.12)	0.91(0.74,1.13)	0.77(0.63,0.96)*	0.88(0.70,1.09)	1.00(0.80,1.23)	0.80(0.65,0.99)*
High school/Junior college	0.88(0.65,1.18)	1.03(0.84,1.25)	0.97(0.80,1.17)	1.02(0.83,1.24)	1.00(0.82,1.23)	0.98(0.79,1.22)	1.06(0.85,1.31)	0.61(0.49,0.75)**	0.82(0.66,1.02)	1.02(0.82,1.27)	0.92(0.74,1.15)
University	0.57(0.40,0.81)*	0.92(0.75,1.13)	0.92(0.75,1.13)	1.06(0.87,1.30)	1.00(0.81,1.23)	1.02(0.81,1.27)	1.15(0.92,1.43)	0.66(0.53,0.82)**	1.01(0.81,1.27)	1.25(1.00,1.57)*	1.15(0.92,1.44)
Master's degree and above	0.67(0.39,1.13)	1.10(0.83,1.46)	0.98(0.75,1.29)	1.12(0.86,1.46)	1.26(0.96,1.67)	1.05(0.79,1.41)	1.11(0.82,1.50)	0.76(0.56,1.02)	1.08(0.81,1.46)	1.35(1.01,1.81)*	1.31(0.97,1.76)
**Household registration**											
Non-rural	Ref.	Ref.	Ref.	Ref.	Ref.	Ref.	Ref.	Ref.	Ref.	Ref.	Ref.
Rural	1.29(1.04,1.61)*	1.01(0.91,1.13)	1.01(0.91,1.12)	0.93(0.84,1.03)	0.95(0.85,1.05)	1.11(0.99,1.24)	1.10(0.98,1.24)	1.01(0.90,1.14)	0.95(0.85,1.06)	1.03(0.93,1.14)	1.07(0.96,1.19)
**Occupation**											
Employed	Ref.	Ref.	Ref.	Ref.	Ref.	Ref.	Ref.	Ref.	Ref.	Ref.	Ref.
Retirement	1.56(1.20,2.04)*	1.16(0.97,1.39)	1.29(1.08,1.53)*	1.19(1.00,1.41)	1.13(0.93,1.36)	1.18(0.98,1.43)	1.21(1.00,1.46)	1.22(1.00,1.49)	0.88(0.72,1.07)	1.01(0.84,1.22)	0.85(0.70,1.03)
Student	1.47(0.78,2.79)	1.13(0.89,1.44)	1.19(0.95,1.50)	1.02(0.80,1.29)	1.25(0.98,1.58)	1.47(1.16,1.87)*	1.19(0.93,1.53)	1.07(0.83,1.37)	0.89(0.69,1.13)	0.94(0.75,1.18)	1.27(1.00,1.60)
Unemployed	1.57(1.17,2.11)*	1.37(1.13,1.66)*	1.46(1.21,1.77)**	1.21(1.00,1.46)*	1.32(1.09,1.60)*	1.27(1.03,1.55)*	1.14(0.93,1.41)	1.10(0.89,1.35)	0.81(0.66,1.00)	0.96(0.78,1.18)	1.05(0.85,1.29)
Others	0.48(0.25,0.93)*	0.71(0.52,0.95)*	0.64(0.47,0.87)*	0.82(0.60,1.12)	0.61(0.44,0.83)*	1.00(0.74,1.35)	0.76(0.55,1.06)	0.90(0.66,1.23)	0.83(0.62,1.12)	0.76(0.57,1.02)	0.69(0.51,0.93)*
**Income/month, RMB**											
0 ∼ 1300	Ref.	Ref.	Ref.	Ref.	Ref.	Ref.	Ref.	Ref.	Ref.	Ref.	Ref.
1300–3300	0.66(0.50,0.86)*	1.01(0.88,1.16)	1.07(0.93,1.22)	0.88(0.77,1.01)	0.88(0.77,1.01)	0.96(0.84,1.11)	0.92(0.80,1.06)	0.82(0.70,0.95)*	0.88(0.76,1.02)	0.93(0.81,1.07)	0.89(0.78,1.03)
3300–6300	1.00(0.74,1.35)	1.00(0.84,1.18)	1.10(0.94,1.30)	0.89(0.76,1.04)	0.99(0.84,1.17)	0.98(0.82,1.16)	0.99(0.84,1.18)	1.02(0.85,1.22)	0.90(0.76,1.07)	1.00(0.85,1.18)	0.93(0.79,1.09)
6300–13000	1.01(0.70,1.47)	0.99(0.81,1.20)	0.97(0.80,1.17)	0.77(0.64,0.93)*	0.94(0.77,1.15)	0.99(0.81,1.22)	0.78(0.63,0.97)*	0.97(0.78,1.19)	0.82(0.66,1.00)	0.83(0.68,1.01)	0.82(0.68,1.00)*
13000–21000	1.34(0.75,2.40)	1.15(0.84,1.57)	1.26(0.92,1.72)	0.67(0.50,0.91)*	1.17(0.87,1.58)	1.05(0.78,1.43)	1.15(0.83,1.60)	1.16(0.84,1.58)	1.14(0.83,1.57)	1.04(0.78,1.39)	1.12(0.83,1.52)
21000–42000	1.46(0.67,3.18)	0.71(0.43,1.18)	1.32(0.82,2.12)	0.79(0.51,1.23)	0.73(0.47,1.15)	0.94(0.59,1.51)	0.66(0.40,1.08)	0.65(0.39,1.08)	1.02(0.63,1.64)	0.88(0.56,1.38)	0.80(0.49,1.31)
42000 and above	0.97(0.53,1.78)	0.90(0.62,1.31)	0.99(0.67,1.47)	0.79(0.56,1.11)	0.73(0.51,1.05)	0.94(0.65,1.36)	0.99(0.67,1.45)	0.94(0.66,1.35)	0.84(0.56,1.25)	1.01(0.68,1.50)	0.78(0.54,1.14)
**Smoking**											
Never smoked	Ref.	Ref.	Ref.	Ref.	Ref.	Ref.	Ref.	Ref.	Ref.	Ref.	Ref.
Occasional smoker	2.83(2.10,3.83)**	1.29(1.08,1.55)*	1.38(1.16,1.64)**	1.00(0.84,1.19)	1.26(1.04,1.51)*	1.26(1.04,1.53)*	1.23(1.01,1.51)*	1.27(1.03,1.56)*	1.24(1.02,1.51)*	1.14(0.94,1.37)	1.20(0.99,1.45)
Frequent smoker	1.55(1.14,2.11)*	1.13(0.95,1.34)	1.24(1.05,1.46)*	0.92(0.78,1.09)	1.12(0.95,1.33)	0.97(0.81,1.16)	1.20(1.00,1.44)	1.09(0.91,1.31)	1.22(1.02,1.46)*	1.20(1.01,1.42)*	1.00(0.84,1.19)
Former smoker	1.05(0.69,1.61)	0.98(0.76,1.26)	1.18(0.91,1.52)	1.05(0.83,1.32)	1.07(0.83,1.38)	0.97(0.75,1.26)	1.35(1.02,1.78)*	1.13(0.86,1.48)	1.31(0.99,1.73)	1.28(0.99,1.65)	1.15(0.88,1.50)
**Drinking**											
Never drink	Ref.	Ref.	Ref.	Ref.	Ref.	Ref.	Ref.	Ref.	Ref.	Ref.	Ref.
Occasional drinker	1.13(0.90,1.43)	1.08(0.96,1.20)	1.14(1.02,1.27)*	1.30(1.17,1.44)**	1.18(1.06,1.32)*	1.30(1.16,1.45)**	1.22(1.09,1.38)*	1.33(1.19,1.50)**	1.43(1.28,1.61)**	1.33(1.19,1.49)**	1.27(1.14,1.42)**
Frequent drinker	2.11(1.52,2.94)**	1.30(1.06,1.61)*	1.43(1.17,1.75)*	1.56(1.27,1.91)**	1.19(0.96,1.48)	1.58(1.28,1.97)**	1.28(1.02,1.60)*	1.99(1.58,2.52)**	1.39(1.11,1.74)*	1.48(1.19,1.84)*	1.29(1.04,1.60)*
Former drinker	1.33(0.90,1.96)	1.18(0.94,1.49)	1.42(1.13,1.78)*	1.31(1.05,1.64)*	1.33(1.05,1.68)*	1.23(0.97,1.55)	1.23(0.96,1.57)	1.45(1.15,1.84)*	1.68(1.33,2.13)**	1.21(0.96,1.52)	1.43(1.13,1.81)*
**Participation in physical activities**											
Frequent participation	Ref.	Ref.	Ref.	Ref.	Ref.	Ref.	Ref.	Ref.	Ref.	Ref.	Ref.
Occasional participation	1.29(1.03,1.61)*	1.43(1.28,1.60)**	1.43(1.29,1.59)**	1.27(1.15,1.41)**	1.44(1.29,1.61)**	1.29(1.15,1.45)**	1.32(1.18,1.49)**	1.31(1.16,1.48)**	1.47(1.31,1.65)**	1.32(1.18,1.47)**	1.31(1.18,1.47)**
Never participate	1.75(1.39,2.21)**	1.43(1.25,1.63)**	1.67(1.46,1.90)**	1.29(1.13,1.46)**	1.79(1.57,2.05)**	1.25(1.09,1.43)*	1.31(1.14,1.50)**	1.25(1.09,1.44)*	1.42(1.23,1.64)**	1.29(1.13,1.48)**	1.31(1.14,1.50)**
Uncertain	2.13(1.40,3.26)**	1.32(0.98,1.77)	1.27(0.97,1.66)	1.19(0.91,1.55)	1.23(0.91,1.65)	1.34(1.02,1.78)*	1.32(0.99,1.76)	1.19(0.89,1.58)	0.95(0.71,1.27)	1.01(0.79,1.30)	1.19(0.91,1.56)
**Changes in self-perceived health status compared to the previous year**											
No change	Ref.	Ref.	Ref.	Ref.	Ref.	Ref.	Ref.	Ref.	Ref.	Ref.	Ref.
Improved	2.05(1.62,2.60)**	0.93(0.82,1.04)	1.01(0.90,1.13)	0.98(0.88,1.10)	0.95(0.85,1.07)	1.31(1.16,1.48)**	1.48(1.31,1.68)**	1.32(1.16,1.50)**	1.03(0.91,1.16)	1.11(0.98,1.24)	1.11(0.98,1.24)
Worsened	2.57(2.05,3.23)**	1.95(1.70,2.23)**	2.10(1.84,2.39)**	2.77(2.43,3.15)**	2.88(2.51,3.30)**	2.87(2.50,3.29)**	3.03(2.64,3.48)**	3.20(2.79,3.68)**	3.49(3.01,4.04)**	2.57(2.24,2.95)**	3.09(2.69,3.55)**
Uncertain	1.50(1.13,2.01)*	1.47(1.28,1.69)**	1.75(1.52,2.00)**	1.80(1.58,2.05)**	1.87(1.63,2.15)**	2.00(1.74,2.31)**	2.29(1.98,2.65)**	2.21(1.91,2.57)**	2.02(1.74,2.34)**	2.17(1.89,2.50)**	1.98(1.73,2.28)**
**Presence of chronic diseases**											
No	Ref.	Ref.	Ref.	Ref.	Ref.	Ref.	Ref.	Ref.	Ref.	Ref.	Ref.
Yes	4.23(3.42,5.24)**	1.70(1.53,1.88)**	1.70(1.54,1.89)**	1.70(1.54,1.88)**	1.98(1.79,2.20)**	2.33(2.10,2.59)**	2.31(2.07,2.57)**	2.68(2.41,2.99)**	2.21(1.97,2.48)**	1.62(1.46,1.80)**	1.79(1.61,1.99)**
**-2 Log L**	1327.09	600.02	768.19	866.24	951.32	961.33	881.51	1256.98	971.14	665.32	840.30
**Pseudo R2**	0.2534	0.0497	0.0541	0.0541	0.0730	0.0832	0.0828	0.1206	0.0808	0.0537	0.0701

*Note*: **indicates *P* < 0.001, *indicates *P* < 0.05

## Discussion

To the best of our knowledge, this study is the first to estimate population norms for the descriptive section of the CQ-11D questionnaire among a representative sample of China. Norms for the CQ-11D were obtained through the usage of a recently developed CQ-11D value set [28]. The CQ-11D is a life quality assessment standard developed based on the principles of traditional Chinese medicine. There exist disparities in the dimensional count between the CQ-11D instrument and the internationally recognized GPBMs such as EQ-5D-5 L and SF-6D. However, comparative studies have demonstrated consistency in the measurement outcomes of these three instruments [32]. The CQ-11D captures aspects of Chinese culture and TCM theory that are not included in other GPBMs (such as appetite, stool, and dizziness). According to the value set of CQ-11D, the largest decrements in utility were observed in the dimensions of Action and life self-care (HD), Pain (TT), Anxiety or depression (JL), and Appetite (SY), which had a significant impact on utility values but were not fully captured by GPBMs [28]. Therefore, the CQ-11D instrument can comprehensively reflect the health preferences and characteristics of the Chinese population, making it more culturally applicable.

Overall, the population had a mean CQ-11D utility score of 0.897(SD: 0.142), which was between the health utility values of the Chinese population measured by EQ-5D (0.946) [20], EQ-5D (0.939) [21], SF-6Dv2 (0.827) [20], SF-6Dv2 (0.872) [21] in previous studies. Similarly, our findings align with other Chinese population norms, indicating a consistent trend. The XD dimension is the least reported of any problem. About 47% of respondents reported any problem and about 3% of respondents reported 3–4 level problems in the TT dimension. It is similar to the reported problem in the pain dimension of SF-6Dv2 in previous studies [20]. The SHEN dimensions (PL, FZ, and JL) problem were more prevalent among the younger population, and similar findings in the EQ-5D-5 L of the Chinese norm indicated that the anxiety/depression problem was more prevalent in the younger population [23]. The potential explanation lies in the fact that the younger generation is exposed to a faster-paced and more stressful urban lifestyle in comparison to the older generation. Subsequently, this may result in higher demands in areas such as employment and education for the younger cohort. Measures such as improving employment security and the employment environment may reduce the pressure on young people and improve their HRQol. Compared with males, females appear to be at an increased risk of reported problems of body and spirit, and relatively lower utilities, which has been similarly found in previous studies [21, 23, 44–46]. In addition, similar findings to other studies were that lower socio-demographic status was associated with poorer HRQoL, i.e. lower income [44], primary education [43], rural householding [47] and so on.

There are also some new findings in this study. The PL dimension was the most frequently reported any problem (2–4 level) and the SM dimension was the most frequently reported 3–4 level (often or severity) problem in our study. Sleep quality and appetite are two health behaviors that were strongly associated with HRQoL [25]. A previous study indicated that sufficient sleep (7–8 h/day) was significantly associated with increased HRQoL [48]. The SM and SY of coefficients of the CQ-11D utility value set were − 0.051∼−0.149, which had a greater impact on the CQ-11D index score. Some previous studies did not find that smoking and drinking had a significant influence on HRQoL [25, 49]. However, smoking and drinking have consistently been recognized as risk factors for numerous chronic diseases, and have also shown their influence on HRQoL in other countries [50, 51].

The findings in this study confirmed that smoking and drinking behaviors influence on HRQoL of the Chinese population. Occasionally/often smoking (OR 1.55 ∼ 2.83) and occasionally/often drinking (OR 1.14 ∼ 2.83) increase the odds of reporting problems in almost all of the dimensions. Besides, compared to non-smokers, quit-smoking respondents had lower odds of reporting problems across all dimensions than those who still smoked, although it was not a significant improvement in CQ-11D index scores. Similar findings were found among respondents who quit drinking and those who still drink alcohol. This finding implies that supporting smokers and drinkers in quitting these behaviors will improve their HRQoL. Participation in physical activities was positively correlated with the health utility value, and that was better when participating in physical exercise regularly. Although occasional physical activity and non-physical activity have similar odds of reporting problems in all dimensions compared with regular physical activity, the former ‘s health utility value is better than the latter. Participating in physical activity, whether regularly or occasionally, is beneficial for CQ-11D index scores, with regular physical activity demonstrating a more pronounced improvement in both physical and mental well-being. Therefore, it is advocated that people reduce or quit smoking and drinking, and take more physical activity. This can reduce the risk of reporting problems in aspects of such as sleep, appetite, stool, palpitation, and fatigue, thereby improving their HRQoL.

This study is subject to several limitations that need to be acknowledged and addressed in future research. Firstly, in the sampling process, the sampling of sex and ethnicity was consistent with the proportions of the seventh population census. However, the sample proportions of some populations (15∼24 years, East China, high education level, and rural household registration) are slightly higher than the seventh population census, which may have a certain impact on the study (Table 1). Given that the health utility value tends to be higher among individuals with a younger age and a higher education level, and the impact of health utility value between rural and non-rural household registrations and East China and other Geographical divisions are relatively smaller, sampling bias may lead to inflated health utility values for the overall sample. Secondly, cross-sectional data could not reflect the impact of time factors on HRQoL in different populations in China. In terms of understanding the causal relationship between variables and controlling for unobserved heterogeneity, longitudinal data is needed [23, 52]. Thirdly, the data used in this study did not match the assumptions of homoscedasticity and normality of the estimation errors. However, some studies of the EQ-5D have utilized OLS [44, 53, 54], while others have compared different modeling techniques and recommended the use of OLS [44, 55, 56]. Consequently, we chose to perform OLS regression analysis for this study as well.

## Conclusions

This study reports the first Chinese population norms for the CQ-11D derived using a representative sample of the Chinese general population. Self-reported health status measured by the CQ-11D varies among different socioeconomic groups. In addition to participation in physical activity and the presence of chronic diseases, smoking, and drinking also significantly influence HRQoL.

### Electronic supplementary material

Below is the link to the electronic supplementary material.

Supplementary Material 1

## Data Availability

The datasets used and/or analyzed during the current study are available from the corresponding author upon reasonable request.

## References

[1] The World Health Organization Quality of Life assessment (WHOQOL): position paper from the World Health Organization. Soc Sci Med. 1995;41(10):1403-9. 10.1016/0277-9536(95)00112-k. PMID: 8560308. 10.1016/0277-9536(95)00112-k 8560308

[2] Zhang L, Wang F, Wang L et al. Prevalence of chronic kidney disease in China: a cross-sectional survey.Lancet,2012,379(9818):815–22. 10.1016/S0140-6736(12)60033-6 22386035

[3] Zimmermann IR, Silva MT, Galvao TF, Pereira MG (2017). Health-related quality of life and self-reported long-term conditions: a population-based survey. Braz J Psychiatry.

[4] Karimi M, Brazier J (2016). Health, health-related quality of life, and quality of life: what is the difference?. PharmacoEconomics.

[5] Robert MK, Andrew L, Ries (2007). Quality of life: Concept and Definition. COPD: J Chronic Obstr Pulmonary Disease.

[6] Guyatt GH, Feeny DH, Patrick DL (1993). Measuring health-related quality of life. Ann Intern Med.

[7] Brauer CA, Rosen AB, Greenberg D, Neumann PJ (2006). Trends in the Measurement of Health Utilities in published cost-utility analyses. Value Health.

[8] Dolan P (1997). Modeling valuations for EuroQol health states. Med Care.

[9] Kharroubi SA, Brazier JE, Roberts J, O’Hagan A, Modelling (2007). SF-6D health state preference data using a nonparametric bayesian method. J Health Econ.

[10] Brazier J, Roberts J, Deverill M (2002). The estimation of a preference-based measure of health from the SF-36. J Health Econ.

[11] The EuroQol Group (1990). EuroQol—a new facility for the measurement of health-related quality of life. Health Policy.

[12] Herdman M, Gudex C, Lloyd A (2011). Development and preliminary testing of the new five-level version of EQ-5D (EQ-5D-5L). Qual Life Res.

[13] Thompson AJ, Turner AJ (2020). A comparison of the EQ-5D-3L and EQ-5D-5L. PharmacoEconomics.

[14] Brazier J, Mulhern BJ, Bjorner JB, SF-6Dv2 International Project Group (2020). Developing a new version of the SF-6D health state classification system from the SF-36v2: SF-6Dv2. Med Care.

[15] Poder TG, Fauteux V, He J (2019). Consistency between three different ways of administering the short form 6 dimension version 2. Value Health.

[16] Guillemin F, Bombardier C, Beaton D (1993). Cross-cultural adaptation of health-related quality of life measures: literature review and proposed guidelines. J Clin Epidemiol.

[17] Herdman M, Fox-Rushby J, Badia X (1998). A model of equivalence in the cultural adaptation of HRQoL instruments: the universalist approach. Qual Life Res.

[18] Mao Z, Ahmed S, Graham C, Kind P, Sun YN, Yu CH (2021). Similarities and differences in health-related quality-of-life concepts between the East and the West: a qualitative analysis of the content of health-related quality-of-life measures. Value Health Reg Issues.

[19] Lam CL, Brazier J, McGhee SM (2008). Valuation of the SF-6D health states is feasible, acceptable, reliable, and valid in a Chinese population. Value Health.

[20] Xie S, Wu J, Xie F (2022). Population norms for SF-6Dv2 and EQ-5D-5L in China. Appl Health Econ Health Policy.

[21] Xie S, Wang D, Wu J, Liu C, Jiang W (2022). Comparison of the measurement properties of SF-6Dv2 and EQ-5D-5L in a Chinese population health survey. Health Qual Life Outcomes.

[22] Wu J, Xie S, He X (2021). Valuation of SF-6Dv2 Health states in China using Time Trade-off and discrete-choice experiment with a duration dimension. PharmacoEconomics.

[23] Yang Z, Busschbach J, Liu G (2018). EQ-5D-5L norms for the urban Chinese population in China. Health Qual Life Outcomes.

[24] Deng X, Dong P, Zhang L (2017). Health-related quality of life in residents aged 18 years and older with and without disease: findings from the First Provincial Health Services Survey of Hunan, China. BMJ Open.

[25] Wu H, Han S, Zhang G (2020). Health-related quality of life and determinants in North-China urban community residents. Health Qual Life Outcomes.

[26] Zhu WT, Gao HL, Zhang MP (2022). Development of the Chinese Medicine Life Quality Evaluation Scale. China J Pharm Econ.

[27] China Association of Chinese Medicine. Evaluation scale for quality of life in Chinese medicine: T/CACM1372-2021 [S] Beijing. China Association of Chinese Medicine; 2021. https://www.ttbz.org.cn/StandardManage/Detail/53564/.

[28] Zhu W, Zhang M, Pan J (2023). Valuing Chinese medicine quality of life-11 dimensions (CQ-11D) health states using a discrete choice experiment with survival duration (DCETTO). Health Qual Life Outcomes.

[29] Poór AK, Rencz F, Brodszky V (2017). Measurement properties of the EQ-5D-5L compared to the EQ-5D-3L in psoriasis patients. Qual Life Res.

[30] Jieqi W, Linlin C (2020). Study on the application of General Utility Scale on Chinese Population. Health Econ Res.

[31] Xudong D, Ping Z (2018). Health utility of patients with stroke measured EQ-5D and SF-6D. J Sichuan Univ (Medical Sci Edition).

[32] Zhou J, Xu L, Pan J (2023). A comparative study of Chinese medicine quality of life assessment scale (CQ-11D) and EQ-5D-5L and SF-6D scales based on Chinese population. Qual Life Res.

[33] Brazier JE, Fukuhara S, Roberts J (2009). Estimating a preference-based index from the Japanese SF-36. J Clin Epidemiol.

[34] Cruz LN, Camey SA, Hoffmann JF (2011). Estimating the SF-6D value set for a population-based sample of brazilians. Value Health.

[35] Ferreira LN, Ferreira PL, Pereira LN, Brazier J, Rowen D. A Portuguese value set for the SF-6D [published correction appears in Value Health. 2015;18(8):1162]. Value Health. 2010;13(5):624–630. 10.1111/j.1524-4733.2010.00701.x. 10.1111/j.1524-4733.2010.00701.x 20230545

[36] Szende A, Janssen B, Cabases J (2014). Self-reported Population Health: An International Perspective based on EQ-5D.

[37] Brazier J, Ara R, Azzabi I, Busschbach J, Chevrou-Severac H, Crawford B (2019). Identification, review, and use of health state utilities in cost-effectiveness models: an ISPOR good practices for outcomes research task force report. Value Health.

[38] Jang R, Janssen MFB, Pickard AS (2021). US population norms for the EQ-5D-5L and comparison of norms from face-to-face and online samples. Qual Life Res.

[39] McCaffrey N, Kaambwa B, Currow DC, Ratcliffe J (2016). Health-related quality of life measured using the Eq. 5D-5L: South Australian population norms. Health Qual Life Outcomes.

[40] Grochtdreis T, Dams J, Konig HH, Konnopka A (2019). Health-related quality of life measured with the EQ-5D-5L: estimation of normative index values based on a representative German population sample and value set. Eur J Health Econ.

[41] Grochtdreis T, Dams J, König HH, Konnopka A (2019). Health-related quality of life measured with the EQ-5D-5L: estimation of normative index values based on a representative German population sample and value set. Eur J Health Econ.

[42] Kivits J, Erpelding ML, Guillemin F (2013). Social determinants of health-related quality of life. Rev Epidemiol Sante Publique.

[43] Fan YJ, Feng YJ, Meng Y, Su ZZ, Wang PX (2022). The relationship between anthropometric indicators and health-related quality of life in a community-based adult population: a cross-sectional study in Southern China. Front Public Health.

[44] Huang W, Yu H, Liu C (2017). Assessing Health-related quality of life of Chinese adults in Heilongjiang using EQ-5D-3L. Int J Environ Res Public Health.

[45] Dong WL, Li YC, Wang ZQ (2016). Self-rated health and health-related quality of life among Chinese residents, China, 2010. Health Qual Life Outcomes.

[46] Sun S, Chen J, Johannesson M, et al. Regional differences in health status in China: population health-related quality of life results from the National Health Services Survey 2008. Health Place. 2011;17(2):671–80. 10.1016/j.healthplace.2011.01.007. 10.1016/j.healthplace.2011.01.007 21334961

[47] Li H, Wei X, Ma A, Chung RY (2014). Inequalities in health status among rural residents: EQ-5D findings from household survey China. Int J Equity Health.

[48] Fujikawa A, Suzue T, Jitsunari F, Hirao T. Evaluation of health-related quality of life using EQ-5D in Takamatsu, Japan. Environ Health Prev Med. 2011;16(1):25–35. 10.1007/s12199-010-0162-1. 10.1007/s12199-010-0162-1 PMC2999682 21432214

[49] Song T, Ding YW, Sun Y et al. A population-based study on health-related quality of life among urban community residents in Shenyang, Northeast of China. BMC Public Health. 2015;15:921. Published 2015 Sep 19. 10.1186/s12889-015-2238-8. 10.1186/s12889-015-2238-8 PMC4575423 26386951

[50] Byles J, Young A, Furuya H, Parkinson L (2006). A drink to healthy aging: the association between older women’s use of alcohol and their health-related quality of life. J Am Geriatr Soc.

[51] Soares MF, Ferreira RC, Pazzini CA, Travassos DV, Paiva SM, e Ferreira EF (2015). Individual and collective empowerment and associated factors among Brazilian adults: a cross-sectional study. BMC Public Health.

[52] Hajek A, Brettschneider C, Mallon T (2017). The impact of social engagement on health-related quality of life and depressive symptoms in old age - evidence from a multicenter prospective cohort study in Germany. Health Qual Life Outcomes.

[53] Luo N (2005). Self-reported Health Status of the General Adult U.S. Population as assessed by the EQ-5D and Health Utilities Index. Med Care.

[54] Abdin E, Subramaniam M, Vaingankar JA, Luo N, Chong SA (2013). Measuring health-related quality of life among adults in Singapore: Population norms for the EQ-5D. Qual. Life Res.

[55] Brazier JE, Yang Y, Tsuchiya A, Rowen DL (2010). A review of studies mapping (or cross walking) non-preference based measures of health to generic preference-based measures. Eur J Health Econ.

[56] Longworth L, Yang Y, Young T, Mulhern B, Hernández AM, Mukuria C, Rowen D, Tosh J, Tsuchiya A, Evans P (2014). Use of generic and condition-specific measures of health-related quality of life in NICE decision-making: a systematic review, statistical modeling and survey. Health Technol Assess.

