# Supplementary material for: Assessing health-related quality of life of Chinese population using CQ-11D
Source: Health Qual Life Outcomes. 2024 Apr 19;22:34. doi: 10.1186/s12955-024-02250-1 (PMC11027529; doi:10.1186/s12955-024-02250-1)
Supplement: Supplementary file 1 — Supplementary Material 1 [file 12955_2024_2250_MOESM1_ESM.docx]

**Appendix 1 The CQ-11D utility value set**

**The CQ-11D utility value set**

| Item level | | Description | Coefficient |
| --- | --- | --- | --- |
| XING^1^  (physical, body) | Action and life self-care（XD） |  |  |
|  | 1 | I don’t have any difficulty in taking care of myself in my actions and life, and there is no problem in my daily activities. | 0 |
|  | 2 | I have a little difficulty moving, but I can take care of myself, and my daily activities are a little restricted | -0.083 |
|  | 3 | I have difficulty taking care of myself in both mobility and life, and my daily activities are very restricted | -0.355 |
|  | 4 | I can’t move and take care of myself, and I can’t carry out daily activities | -0.500 |
|  | Appetite  （SY） |  |  |
|  | 1 | My appetite is very good | 0 |
|  | 2 | My appetite is good | 0 |
|  | 3 | My appetite is poor | -0.102 |
|  | 4 | My appetite is very bad | -0.149 |
|  | Stool  （DB） |  |  |
|  | 1 | My stool movements are very good | 0 |
|  | 2 | My stool movements are good | -0.011 |
|  | 3 | My stool movements are poor | -0.060 |
|  | 4 | My stool movements are very bad | -0.099 |
|  | Sleep quality  （SM） |  |  |
|  | 1 | My sleep quality is very good | 0 |
|  | 2 | My sleep quality is good | 0 |
|  | 3 | My sleep quality is poor | -0.051 |
|  | 4 | My sleep quality is very poor | -0.118 |
|  | Vigour  （JS） |  |  |
|  | 1 | My vigour is very good | 0 |
|  | 2 | My vigour is good | -0.022 |
|  | 3 | My vigour is bad | -0.079 |
|  | 4 | My vigour is very bad | -0.143 |
|  | Dizziness  （TY） |  |  |
|  | 1 | I am not dizzy at all | 0 |
|  | 2 | I occasionally feel dizzy | 0 |
|  | 3 | I often feel dizzy | -0.068 |
|  | 4 | I feel dizzy almost every day | -0.135 |
|  | Palpitation  （XH） |  |  |
|  | 1 | I didn't feel palpitations at all | 0 |
|  | 2 | I occasionally feel palpitations | -0.007 |
|  | 3 | I often feel palpitations | -0.045 |
|  | 4 | I feel palpitations almost every day | -0.131 |
|  | Pain  （TT） |  |  |
|  | 1 | I have no pain at all | 0 |
|  | 2 | I have some pain | -0.036 |
|  | 3 | I have severe pain | -0.112 |
|  | 4 | I have very severe pain | -0.211 |
| SHEN^2^  (Spirit, mental) | Fatigue  （PL） |  |  |
|  | 1 | I don't feel tired at all | 0 |
|  | 2 | I occasionally feel a little fatigue | 0 |
|  | 3 | I often feel severe fatigue | -0.060 |
|  | 4 | I feel very tired almost every day | -0.114 |
|  | Irritability  （FZ） |  |  |
|  | 1 | I don't feel irritable at all | 0 |
|  | 2 | I occasionally feel irritable | -0.006 |
|  | 3 | I often feel irritable | -0.040 |
|  | 4 | I feel irritable almost every day | -0.109 |
|  | Anxiety or depression（JL） |  |  |
|  | 1 | I don't feel anxious or depressed at all | 0 |
|  | 2 | I occasionally feel anxious or depressed | 0 |
|  | 3 | I often feel anxious or depressed | -0.052 |
|  | 4 | I feel anxious or depressed almost every day | -0.159 |

Note: ^1^XING, denotes the “body” in the unity between the body and the Shen (Spirit) theoretical of Chinese medicine; ^2^SHEN, denotes the “spirit” in the unity between the body and the Shen (Spirit) theoretical of Chinese medicine.
